# *Amomum tsaoko* extract from Nujiang alleviates DSS-induced colitis through inhibiting necroptosis

**DOI:** 10.3389/fcell.2026.1768630

**Published:** 2026-02-26

**Authors:** Yuanyuan Wang, Keyi Lu, Yuhang Gong, Yanna Shao, Siqi Liu, Yuan Fang, Yifan Shi, Erping Xu, Yanqiong Yang, Si Yuan, Ming Bai, Zhibin Wang, Bo Zhang

**Affiliations:** 1 Collaborative Innovation Center of Research and Development on The Whole Industry Chain of Yu-Yao, Henan Province, Henan University of Chinese Medicine, Zhengzhou, Henan, China; 2 Academy of Chinese Medical Sciences, Henan University of Chinese Medicine, Zhengzhou, Henan, China; 3 Shanghai Municipal Hospital of Traditional Chinese Medicine, Shanghai University of Traditional Chinese Medicine, Shanghai, China; 4 Nujiang Lisu Autonomous Prefecture Traditional Chinese Medicine Hospital, Nujiang, Yunnan, China; 5 Department of Critical Care Medicine, School of Anesthesiology, Naval Medical University, Shanghai, China

**Keywords:** *Amomum tsaoko*, bioinformatics analysis, necroptosis, STAT3, ulcerative colitis

## Abstract

Necroptosis, a form of programmed cell death, plays a significant role in compromising the intestinal barrier and initiating intestinal inflammation. An analysis of data from the Gene Expression Omnibus (GEO) reveals a strong correlation between ulcerative colitis (UC) and the pathological mechanisms of necroptosis. Consequently, inhibiting necroptosis may offer a promising strategy for ameliorating UC. Cao Guo (CG), a traditional Chinese medicine extensively utilized in China for both dietary and medicinal purposes, is frequently included in classical prescriptions for UC treatment. However, the precise chemical constituents of CG and its potential therapeutic targets for UC remain inadequately characterized. In this study, we analyzed the chemical composition of CG, employed a classic necroptosis model to assess the anti-necroptosis activity of CG, and conducted validation using a mouse model of UC. Through bioinformatics analysis and other methodologies, we initially explored the potential targets of CG in UC treatment and conducted subsequent verification. The findings demonstrate that in an *in vitro* necroptosis model, CG significantly enhances cell morphology and survival rates, while concurrently inhibiting the phosphorylation of RIPK1, RIPK3, and MLKL. In a mouse model of UC, CG alleviates weight loss and the disease activity index (DAI), ameliorates intestinal histopathological conditions, and upregulates the expression of tight junction proteins, such as ZO-1 and Occludin. Concurrently, CG diminishes the distribution and expression of phosphorylated RIPK3 and MLKL. Bioinformatics analysis suggests that CG may inhibit necroptosis via the signal transducer and activator of transcription 3 (STAT3) pathway, a hypothesis preliminarily validated through experimental methods. These results indicate that CG may exert therapeutic effects on UC by inhibiting STAT3 phosphorylation and suppressing necroptosis.

## 1 Introduction

Ulcerative colitis (UC) is a form of inflammatory bowel disease (IBD) characterized by persistent chronic inflammation of the colorectum (Ungaro et al., 2017). The clinical symptoms of UC include diarrhea, abdominal pain, and the presence of mucus, pus, and blood in the stool (Honap et al., 2024), with an increasing incidence rate observed annually (Le Berre et al., 2023). Current clinical treatment strategies include the use of corticosteroids, immunosuppressants, biologic agents, and surgical interventions. While these therapeutic approaches can mitigate and alleviate symptoms such as abdominal pain, diarrhea, and bloody stools, the complete restoration of intestinal barrier function remains an urgent concern (Kotla et al., 2022). Consequently, substantial efforts are being directed towards enhancing the quality of life and long-term prognosis for UC patients.

The primary objective in the treatment of UC is the restoration of the compromised intestinal mucosa and mucosal inflammation (Xiao et al., 2016). The aberrant death of intestinal epithelial cells is the most immediate cause of epithelial cell injury (Patankar and Becker, 2020), which subsequently results in the disruption of the tight junction (TJ) barrier (Turner, 2009; Keita and Söderholm, 2010). This disruption increases intestinal permeability, facilitating the infiltration of harmful substances that compromise intestinal integrity and exacerbate the inflammatory response. This inflammatory response, in turn, perpetuates the abnormal death of intestinal epithelial cells, thereby keeping intestinal inflammation and mucosal injury. Consequently, targeting the aberrant death of intestinal epithelial cells may represent a novel therapeutic strategy for UC.

Necroptosis represents a distinct form of programmed cell death, differing from traditional apoptosis by inducing cell swelling, membrane rupture, and the subsequent release of substantial inflammatory mediators (Christofferson and Yuan, 2010). Numerous studies have validated that targeting necroptosis constitutes an effective therapeutic strategy for UC (Zhang et al., 2020; Akanyibah et al., 2024; Shen et al., 2023; Liu et al., 2025; Wang et al., 2024). In the context of necroptosis research, tumor necrosis factor-alpha (TNF-α) is identified as the principal inducer. Upon TNF stimulation, TNF receptor 1 (TNFR1) typically recruits key components, including the TNF receptor-associated death domain (TRADD), TNF receptor-associated factor 2 (TRAF2), cellular inhibitors of apoptosis proteins 1 and 2 (cIAP1/2), and receptor-interacting protein kinase 1 (RIPK1), to form complex I on the plasma membrane, thereby facilitating NF-κB signaling (Chen and Goeddel, 2002; Wajant et al., 2003; Christofferson et al., 2014). When Caspase-8 activity is blocked, RIPK1 and receptor-interacting protein kinase 3 (RIPK3) interact via the RIP homotypic interaction motif, promoting the necroptotic pathway (O'Donnell et al., 2011; Feoktistova et al., 2011). The formation of RIPK1-RIPK3 necrosomes activates Mixed Lineage Kinase Domain-Like Protein (MLKL), which subsequently undergoes oligomerization and translocates to the cell membrane (Dondelinger et al., 2014; Cai et al., 2014). This process results in cell membrane rupture and the induction of necroptosis. Following membrane rupture, substantial quantities of cellular contents and damage-associated molecular patterns (DAMPs) are released. The excessive loss of intestinal epithelial cells, coupled with inflammation and damage to the intestinal barrier, creates a mutually aggravating relationship, perpetuating the exacerbation of intestinal inflammation.

Based on clinical manifestations such as persistent or recurrent diarrhea, mucoid bloody stool, abdominal pain, and abdominal distension, UC is classified within the category of diarrhea or dysentery in traditional Chinese medicine (TCM). The pathogenesis of UC is intricate, involving both deficiency and excess, and is associated with cold and dampness. In recent years, there has been a growing interest in TCM and natural products. TCM has shown relatively distinct therapeutic efficacy, with fewer adverse effects and a reduced recurrence rate in the management of UC. Our previous research has shown that the traditional Chinese herb *Sargentodoxa cuneata* (Da Xueteng) (Wang et al., 2024), along with natural products such as gambogic acid (Wang et al., 2025) and pristimerin (Liu et al., 2025), demonstrates significant anti-UC properties via inhibiting necroptosis.

*Amomum tsaoko* Crevost & Lemaire. (Cao Guo, CG), a traditional Chinese herbal medicine with both culinary and therapeutic uses, the first record of CG was in originates from the book of *Yinshan Zhengyao*. CG possesses a pungent and warm nature and enters the spleen and stomach meridians, which enables it to effectively alleviate dampness and provide warmth to the body’s core. It is frequently employed as a seasoning in Chinese households, especially for meat stews. Recent studies have confirmed the therapeutic potential of CG, including anti-microbial, anti-viral, anti-oxidant, anti-obesity, anti-inflammatory, and anti-proliferative activities (Lee et al., 2019; Imran et al., 2024; Li et al., 2014; Yang et al., 2010; Cui et al., 2017; Dai et al., 2016). The CaoGuo-4 decoction, with CG as its principal component, is utilized to address diarrhea resulting from spleen and stomach yang deficiency (Mei et al., 2022). The flavonoid extract derived from CG has been shown to ameliorate UC symptoms (Huang et al., 2025). However, the active components and mechanisms underlying its efficacy in treating UC remain insufficiently elucidated.

This study aims to explore the effects and active constituents of CG in the treatment of UC, with a particular emphasis on its role in necroptosis. Our findings demonstrate that CG possesses significant anti-necroptotic properties both *in vivo* and *in vitro*. Through the application of ultra-performance liquid chromatography quadrupole time-of-flight mass spectrometry (UPLC-QTOF-MS) combined with bioinformatics analysis, we identified the signal transducer and activator of transcription 3 (STAT3) as a potential target for the anti-UC effects of Cao Guo, mediated through the inhibition of necroptosis.

## 2 Materials and methods

### 2.1 Identification of the components of CG

#### 2.1.1 Cao Guo (CG)

The herb *Amomum tsaoko* donated by Dr. Yanqiong Yang. According to the quality control criteria specified in the Chinese Pharmacopoeia (2020), a clinical dose of 6 g per day for humans was utilized, alongside the human-to-animal surface area ratio, to determine the equivalent dose (He et al., 2024). As a result, the dosage of CG employed in this study was calculated to be 0.91 g/kg. Furthermore, to determine a more appropriate dosage, a high concentration—defined as twice the standard dose—was established at 1.82 g/kg. For the *in vivo* experiments, the CG concentration was diluted to 182/91 mg/mL with water, and a dose of 200 μL was administered per mouse. The administration frequency of CG is once a day.

#### 2.1.2 Preparation of freeze-dried CG powder

The CG herb was initially steeped in distilled water at a tenfold volume for 2 hours, followed by a boiling period of 40 minutes. A subsequent decoction was prepared using an eightfold volume of distilled water, following the same procedure. The final extract was produced by combining the two decoctions, which were then filtered through gauze and distilled. After a 48-hour drying period under vacuum conditions, the CG was freeze-dried to obtain a powdered form. The extraction yield was determined to be 13.3%.

#### 2.1.3 Chromatographic/mass spectrometry conditions

The chemical analysis, prototype, and metabolite components of CG were performed utilizing a Waters H-Class UPLC System (Waters Technology Co., Ltd, Milford, USA) in combination with Sciex Triple TOF® 4600 high-resolution mass spectrometry (AB Sciex, Darmstadt, Germany). The chromatographic separation was achieved using a Waters ACQUITY UPLC CSH C18 column (2.1 × 100 mm, 1.7 µm). The mobile phase consisted of water with acetonitrile (solvent A) and 0.1% formic acid (solvent B). The gradient elution was programmed as follows: 0–3 min, 3% A and 97% B; 5–10 min, 3%–10% A and 97%–90% B; 7–20 min, 10%–20% A and 90%–80% B; 20–30 min, 20%–50% A and 80%–50% B; 30–40 min, 50%–90% A and 50%–10% B; 40–44 min, 90% A and 10% B; 44–44.1 min, 90%–3% A and 10%–97% B; 44.1–48 min, 3% A and 97% B. The column oven temperature was maintained at 30 °C, with a flow rate set at 0.3 mL/min. Mass spectrometric detection was conducted in both negative and positive ion modes using an electrospray ionization source.

#### 2.1.4 Data analysis strategy

In this study, data collection was facilitated by the use of Analyst TF 1.7.1 software, while data analysis was conducted using Peakview 1.2. During the identification process, mass spectrometry data were initially compared against the Natural Products HR-MS/MS Spectral Library 1.0 database. Subsequently, the compounds underwent a preliminary evaluation based on the score data associated with each chromatographic peak. Further verification was performed by examining both the primary and secondary data corresponding to each chromatographic peak. The Natural Products HR-MS/MS Spectral Library 1.0 database includes multistage mass spectrometry data derived from our standard products as well as other standard products. This encompasses various collection modes, admixtures, collision energies, and additional parameters, resulting in a comprehensive repository of compound information. The database exclusively contains authentic maps of the collected standard products, devoid of any simulated speculation, thereby ensuring highly accurate matching results. Compounds not present in the database were identified through literature reports and adherence to established mass spectrum fragmentation rules.

### 2.2 Bioinformatics

#### 2.2.1 Collection and organization of GEO data

High-throughput genomic data from the blood samples of patients diagnosed with clinical ulcerative colitis were systematically retrieved and analyzed using the GEO database. Following a rigorous screening process, GSE193677 and GSE186507 were identified and selected for further study. Each dataset comprised samples from both healthy volunteers and UC patients, with a minimum of three samples per group. Subsequent to data collection, gene expression data were exported, and Gene Symbol conversion was conducted utilizing the DAVID platform. Our aim was to identify differentially expressed genes and enriched pathways associated with the systemic inflammatory state of UC, thereby providing insights and directions for subsequent investigations into tissue-specific mechanisms using animal models.

#### 2.2.2 Data acquisition and preprocessing

For the RNA-seq datasets (GSE193677 and GSE186507), the data acquisition involved downloading the original gene count matrix from the GEO database. In the preprocessing stage, we employed the ggplot2 package in R to generate box plots for visual inspection, and utilized the normalizeBetweenArrays function from the limma package for normalization. Differential expression analysis was subsequently conducted on this standardized data. Differentially expressed genes between the subject and control groups in each dataset were identified using the limma package, applying a filtering threshold of LogFC >1 and P-value <0.05. We then applied the RobustRankAggreg (RRA) method to integrate genes that were consistently upregulated or downregulated across both datasets.

#### 2.2.3 Functional enrichment analysis and construction of protein-protein interaction (PPI) network

ClusterProfiler was used to study gene distribution in key modules via GO and KEGG analyses. After identifying the target module, intersections of all key genes in the module with CG (Supplementary Table S2) and necroptosis-related genes (Supplementary Table S3) were determined. Protein interaction network analysis was performed using PPI through the STRING database, leading to the identification of potential targets for treating ulcerative colitis by adding CG to inhibit necroptosis.

### 2.3 Experimental verification

#### 2.3.1 Reagents

Necrostain-1 (Nec-1) (S8037) was acquired from Selleck. Calcein-AM/PI Cell Viability/Cytotoxicity Assay Kit was bought from Beyotime (C2015M). CellTiter-Glo Luminescent Assay was bought from Beyotime (C0065L). Human-TNF-alpha (C008) was purchased from Novoprotein. SM-164 Hydrochloride (HY-15989A) was acquired from Med Chem Express. Z-VAD-fmk (T6013) was bought from TargetMol. Anti-RIPK1 (3493S) and human-specific anti-phospho-RIPK1 (65746S) was bought from Cell Signaling Technology. The BCA protein assay kit (CW0014S) was acquired from CWBIO. The RIPK3 polyclonal antibody (17563-1-A) and Anti-MLKL (66675-1-Ig) were from Proteintech. Anti-RIPK3/p-RIPK3 (ab209384), anti-MLKL (ab184718), human-specific anti-phospho-MLKL (ab187091) and mouse-specific anti-phospho-RIPK3 (ab195117) were bought from Abcam. The mouse-specific anti-phospho-MLKL (bsm-54104R) came from Bioss (Beijing, China). The mouse-specific anti-phospho-RIPK1 (BX60008) was bought from Biolynx (Hangzhou, China). The Occludin (GB111401), p-STAT3 (GB150001) and ZO-1 (GB111402) antibodies were supplied by Servicebio (Wuhan, China). Anti-STAT3 came from Selleck (F0200). Dextran sulfate sodium (DSS) (MW: 36,000–50,000, cat#CD4421) was bought from Coolaber (Beijing, China). The Anti-GAPDH (ab181602) was purchased from Abcam. The TUNEL kit (G1502) was supplied by Servicebio. The HRP Goat anti-rabbit IgG (abs20040) was bought from Absin. The high-intensity ECL Western blotting substrate was from Tanon.

#### 2.3.2 Cell culture

The HT-29 cell line (Procell, CL-0118) was obtained from Procell Life Science & Technology Co., Ltd. The cells were cultured at 37 °C with 5% CO_2_ in growth medium (Procell, CM-0118) supplemented with 10% fetal bovine serum (BI, C04001-500) and 100 U/mL penicillin/streptomycin (Gibco, 15140-122).

#### 2.3.3 Anti-necroptosis activity of CG

HT-29 cells were seeded in 96-well plates at 10,000 cells per well, with triplicates for each group. After 12 h, freeze-dried CG powder was diluted to final concentrations ranging from 100–1.5 mg/mL with pre-treated with 10 nM SM-164 hydrochloride and 20 μM caspase inhibitor z-VAD-fmk for 30 min. Then the cells were stimulated with 20 ng/mL h-TNF-α for another 12 h (TSZ, a combination of h-TNF-α, SM-164, and Z-VAD-FMK). Cell viability was measured with the CellTiter-Glo Luminescent Assay and luminescence was recorded using a SpectraMax M5 (Molecular Devices).

#### 2.3.4 Double staining of live/dead cells

HT-29 cells were collected in accordance with previously established protocols, and 100 μL of a Calcein-AM/PI working solution (dilution 1:1000) was subsequently introduced to each well after a 12-h incubation period. The plates were then incubated for an additional 30 min at 37 °C, protected from light. Cellular viability within each group was evaluated using a microscope (Nikon, Tokyo, Japan), with red fluorescence signifying non-viable cells and green fluorescence signifying viable cells.

#### 2.3.5 Animals

Forty male C57BL/6J mice (6–7 weeks old, 21–23 g) were sourced from Liaoning Changsheng Biotechnology Co., Ltd. They were kept at 23 °C–25 °C, 50%–60% humidity, with a 12-h light/dark cycle, and given unlimited access to standard rodent chow and tap water. The Animal Care and Use Committee of Henan University of Chinese Medicine approved all procedures (approval no. DWLLSZR202508058; Henan, China).

#### 2.3.6 UC model induced by DSS

Male C57BL/6J mice were allocated into five distinct experimental groups: the control group (Con), the DSS-treated group (DSS), the low-dose CG group (CG-L 0.91 g/kg), the high-dose CG group (CG-H 1.82 g/kg), and the Nec-1 group (5 mg/kg), with each group comprising eight mice. Based our approach on both our previous research and existing literature. The final dosage of Nec-1 is set at 5 mg/kg, with a solvent composition of 5% DMSO and 95% physiological saline. The method of administration is intraperitoneal injection, with a frequency of once per day. The control group was maintained on a standard diet and provided with water, whereas the remaining groups received 2.5% DSS for a duration of 7 days to induce UC. The administration period for DSS spans from day 0 to day 7, after which the administration commences. The administration of CG and Nec-1 was from the 8th day to the 13th day. On the 13th day of the study, all animals were humanely euthanized by certified personnel via the administration of an overdose of sodium pentobarbital (150 mg/kg, administered intraperitoneally). Following the induction of unconsciousness, cervical dislocation was performed as a secondary measure to ensure the complete cessation of cerebral activity. The confirmation of death for each animal was established by the absence of detectable cardiac activity and the loss of corneal reflexes. All animal procedures were conducted at the Animal Experimental Center of Henan University of Chinese Medicine. Post-mortem, colonic tissue samples were collected for further analysis.

#### 2.3.7 Disease activity index (DAI)

The DAI score is derived from the aggregation of three distinct parameters: body weight loss, presence of blood in stool, and severity of diarrhea. Body weight loss is evaluated on a scale ranging from 0, indicating no loss, to 4, representing a loss exceeding 20%. The presence of blood in stool is similarly assessed, with a score of 0 denoting no blood and a score of 4 indicating gross bleeding. Diarrhea severity is categorized as follows: 0 for normal consistency, 1 for soft stools, 2 for soft and pasty stools, 3 for stools that are soft and pasty and adhere to the anus, and 4 for liquid stools. The cumulative DAI score is calculated by summing the scores from these individual parameters.

#### 2.3.8 Hematoxylin and eosin (H&E) staining

Mouse colon tissues underwent fixation, dehydration, and embedding into sections, followed by deparaffinization in water. Subsequently, the sections were stained with hematoxylin for 3–5 min at room temperature, rinsed, and blued. Thereafter, the sections were dehydrated in 95% alcohol for 1 min and counterstained with eosin for 15 s at room temperature. Finally, the sections were further dehydrated, mounted, and cover slipped in preparation for imaging and analysis.

#### 2.3.9 AB-PAS staining

Mouse colon tissues underwent fixation, dehydration, and embedding into sections, followed by deparaffinization in water. Subsequently, the slides were sequentially stained with AB-PAS staining solutions C, B, and A, followed by sealing with neutral gum. Micro-microscopic examination, image acquisition and analysis.

#### 2.3.10 TUNEL staininng

Paraffin-embedded sections of mouse colon tissue underwent deparaffinization to water, followed by a 20-minute incubation at 37 °C with a proteinase K working solution to facilitate antigen retrieval. The sections were then subjected to three washes with phosphate-buffered saline (PBS) for 5 min each at room temperature. A membrane-permeabilization solution was applied to cover the tissue, followed by a 20-minute incubation at room temperature, and another PBS wash. Subsequently, the sections were incubated in the TUNEL reaction solution for 1 h at 37 °C. The quantity of terminal deoxynucleotidyl transferase (TDT) enzyme from the TUNEL kit (catalog number G1502; Wuhan Servicebio Technology Co., Ltd.) was adjusted according to the number of slides and tissue size and mixed with dUTP and buffer in a 1:5:50 ratio. After thorough washing with PBS, the sections were stained with DAPI for 10 min at room temperature, protected from light. A final PBS wash was performed, and the sections were mounted using an anti-fluorescence quenching mounting medium. Images were subsequently captured using a fluorescence microscope.

#### 2.3.11 Immunohistochemistry (IHC) staining

Mouse colon tissues were subjected to fixation, dehydration, and embedding into sections, followed by deparaffinization in water. Subsequently, antigen retrieval was performed, followed by the inhibition of endogenous peroxidase activity and serum blocking. The sections were then incubated with a primary antibody, followed by a secondary antibody. DAB staining was applied, and the cell nuclei were counterstained. Finally, the sections underwent dehydration and sealing. The results were interpreted using a white light microscope.

#### 2.3.12 Immunofluorescence staining

Paraffin-embedded intestinal tissue sections were cut into slices. After removing the paraffin, the epithelial monolayer was immunostained. Slides were incubated overnight with antibodies at 4 °C, rinsed with PBS four times, and then incubated with a secondary antibody for 1 h in the dark. They were stained with DAPI (G1012, Servicebio, China) for 10 min at room temperature, rinsed with PBS, and treated with an anti-fluorescence quencher. Following the primary antibodies, Affini Pure goat anti-rat IgG (1:200) (GB21303, Servicebio, China) and goat anti-mouse IgG (1:200) (GB22301, Servicebio, China) were used as secondary antibodies. The fluorescent images of samples were captured using confocal microscopy.

#### 2.3.13 Western blot

HT-29 cells were cultured in 6-well plates at a density of 100,000 cells per well and subjected to treatment with varying concentrations of CG or stimulation with TSZ for different time intervals to evaluate their anti-necroptosis activity. Post-treatment, cell lysis was performed using RIPA Lysis Buffer (CWBIO, Jiangsu, China), and protein concentrations were quantified utilizing a BCA protein assay kit (CWBIO, Jiangsu, China). The protein extracts were then resolved by 10% SDS-PAGE and transferred onto PVDF membranes (Millipore, Bedford, MA, USA). The membranes were blocked with 5% BSA for 2 h and subsequently incubated overnight at 4 °C with various primary antibodies at a 1:1000 dilution. Following a 1-hour incubation with HRP-conjugated secondary antibodies, the membranes were analyzed using an imaging system (Bio-Rad, Hercules, USA), and band optical densities were quantified using ImageJ software.

#### 2.3.14 STAT3 activation

To investigate the potential active role of STAT3, the known STAT3 activator Colivelin was employed. HT-29 cells were treated with 50 mg/mL of CG, both with and without the presence of Colivelin (0.4 μM), within a TSZ-induced necroptosis model. Cell viability was assessed using the CellTiter-Glo Luminescent Assay, and luminescence was recorded utilizing a SpectraMax M5 (Molecular Devices).

#### 2.3.15 Statistical analysis

For longitudinal data collected at multiple time points, such as daily weight or disease activity index, repeated measures analysis of variance (ANOVA) is employed for statistical evaluation. Initially, the Mauchly’s test of sphericity is performed. If the assumption of sphericity is violated (p < 0.05), the Greenhouse-Geisser correction is applied to adjust the degrees of freedom. In the analysis model, “treatment” is considered as the between-subjects factor and “time” as the within-subjects factor, with the interaction effect of “treatment × time” being assessed. Upon finding a significant interaction effect, simple effect comparisons are conducted between groups at each time point, with multiple comparisons corrected using the Bonferroni method. For data measured at a single time point, such as colon length or histological score, a one-way ANOVA followed by Tukey’s post-hoc test is utilized. A p-value of less than 0.05 was considered to indicate statistical significance.

## 3 Results

### 3.1 GEO database suggests that UC is related to necroptosis

**FIGURE 1:**
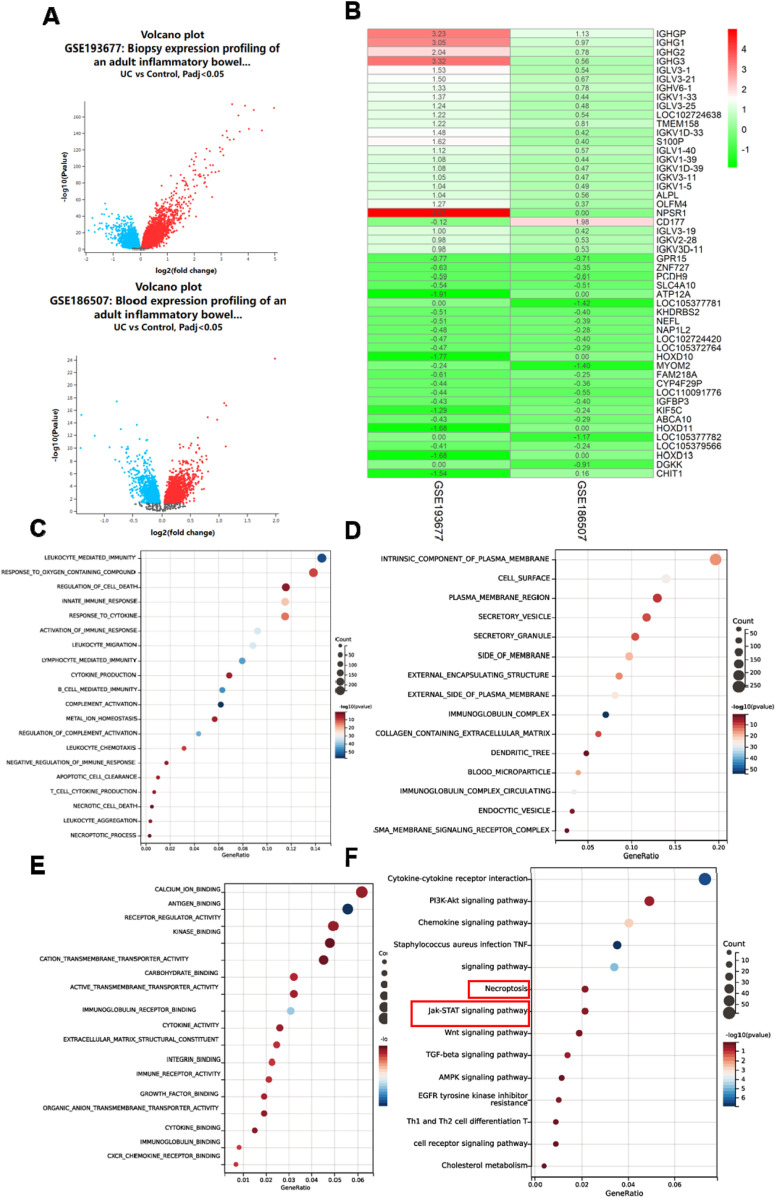
UC is related to necroptosis. **(A)** Volcano plots of all differentially expressed genes in the GSE193677 and GSE186507 datasets. **(B)** Heat map of co-expressed genes in the GSE193677 and GSE186507 datasets. Correlation enrichment analysis of GO-BP **(C)**, GO-CC **(D)**, GO-MF **(E)** and KEGG **(F)** of co-expressed genes in the GSE193677 and GSE186507 datasets.

With “ulcerative colitis” as the main keyword, datasets GSE193677 and GSE186507 were obtained from the GEO database. The gene expression profiles of the UC group in these datasets were compared with those of normal subjects to perform a differential expression analysis, resulting in two corresponding differential expression profiles. The volcano plots illustrating all differentially expressed genes are presented in Figure 1A. Employing the RRA package in R, the differentially upregulated and downregulated genes across the two datasets were integrated to identify co-upregulated and co-downregulated genes. The heatmap depicting these co-expressed genes is shown in Figure 1B. Gene Ontology (GO) (Figures 1C–E) and Kyoto Encyclopedia of Genes and Genomes (KEGG) (Figure 1F) analyses were conducted on the co-upregulated and co-downregulated genes. The results indicate that ulcerative colitis is associated with the necroptosis signaling pathway and the STAT3 signaling pathway.

### 3.2 Component analysis of herb CG

**FIGURE 2:**
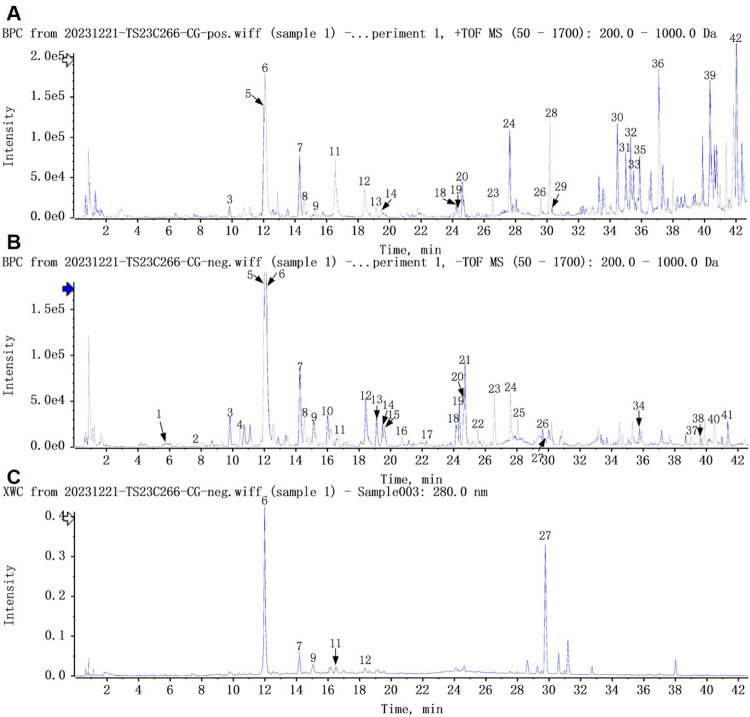
Component analysis of CG using UPLC-Q-TOF/MS. **(A)** Positive ion mode UPLC-HRMS base peak ion flow graph (BPC) for CG; **(B)** Negative ion mode UPLC-HRMS BPC for CG; **(C)** UPLC UV chromatogram of CG at 280 nm.

Freeze-dried CG powder was subjected to analysis using ultra-performance liquid chromatography coupled with high-resolution quadrupole time-of-flight mass spectrometry (UPLC-Q-TOF/MS). The UPLC-Q-TOF/MS chromatographic profile is shown in Figure 2. Utilizing the multi-level mass spectrometric data obtained from the samples, in conjunction with a high-resolution mass spectrometry database of natural products and pertinent literature, a total of 42 compounds were identified from the CG samples, as detailed in Table 1 and Supplementary Table S1.

**TABLE 1:** Identification results of components of CG.

No	Retention time (min)	Adducts	Measured *m/z*	Excepted *m/z*	ppm	Formula	Molecular weight	Phtochemical name
1	5.56	[M-H]^-^	153.0188	153.0188	0.0	C_7_H_6_O_4_	154.03	Protocatechuic acid
2	7.49	[M-H]^-^	137.0247	137.0244	2.2	C_7_H_6_O_3_	138.03	Protocatechuic aldehyde
3	9.82	[M-H]^-^	289.0704	289.0712	−2.8	C_15_H_14_O_6_	290.08	Catechin
4	10.47	[M-H]^-^	451.1340	451.1340	0.0	C_20_H_24_N_2_O_10_	452.14	N-[2-(1-β-D-Glucopyranosyl-1H-indol-3-yl)acetyl]-L-aspartic acid
5	11.99	[M-H]^-^	577.1328	577.1352	−4.2	C_30_H_26_O_12_	578.14	Procyanidin B1
6	12.07	[M-H]^-^	289.0716	289.0712	1.4	C_15_H_14_O_6_	290.08	(-)-Epicatechin
7	14.26	[M-H]^-^	865.1996	865.1985	1.3	C_45_H_38_O_18_	866.21	Procyanidin C1
8	14.55	[M-H]^-^	273.0764	273.0768	−1.5	C_15_H_14_O_5_	274.08	Afzelechin
9	15.13	[M-2H]^2-^	576.1287	576.1273	2.4	C_60_H_50_O_24_	1154.27	Proanthocyanidin tetramer
10	16.02	[M-H]^-^	233.0849	233.0853	−1.7	C_10_H_18_O_4_S	234.09	Geraniol sulfate
11	16.54	[M + H]^+^	597.3274	597.3269	0.8	C_31_H_48_O_11_	596.32	—
12	18.43	[M-H]^-^	577.1340	577.1352	−2.1	C_30_H_26_O_12_	578.14	Procyanidin B2
13	19.12	[M-H]^-^	463.0873	463.0882	−1.9	C_21_H_20_O_12_	464.10	Hyperoside
14	19.49	[M-H]^-^	463.0883	463.0882	0.2	C_21_H_20_O_12_	464.10	Isoquercitrin
15	19.62	[M-H]^-^	605.1666	605.1665	0.2	C_32_H_30_O_12_	606.17	[2R-[2α,3β,8(2R*,3S*)]]-8,8′-ethylidenebis[2-(3,4-dihydroxyphenyl)-3,4-dihydro-2H-1-Benzopyran-3,5,7-triol
16	20.71	[M-H]^-^	577.1347	577.1352	−0.9	C_30_H_26_O_12_	578.14	Procyanidin B3
17	22.48	[M-H]^-^	477.1025	477.1039	−2.9	C_22_H_22_O_12_	478.11	Isorhamnetin-3-O-glucoside
18	24.23	[M-H]^-^	605.1664	605.1665	−0.2	C_32_H_30_O_12_	606.17	(2R,2′R,3R,3′R)-8,8′-ethylidenebis[2-(3,4-dihydroxyphenyl)-3,4-dihydro-2H-1-Benzopyran-3,5,7-triol
19	24.35	[M-H]^-^	315.1600	315.1602	−0.6	C_19_H_24_O_4_	316.17	(+)-Hannokinol
20	24.61	[M + H]^+^	577.1350	577.1341	1.6	C_30_H_24_O_12_	576.13	Procyanidin A1
21	24.71	[M-H]^-^	315.1594	315.1602	−2.5	C_19_H_24_O_4_	316.17	3,5-Dihydroxy-1,7-bis(4-hydroxyphenyl)heptane
22	25.54	[M-H]^-^	313.1442	313.1445	−1.0	C_19_H_22_O_4_	314.15	(5S)-5-Hydroxy-1,7-bis(4-hydroxyphenyl)-3-heptanone
23	26.58	[M-H]^-^	343.1557	343.1551	1.7	C_20_H_24_O_5_	344.16	rel-(2R,3S)-1,4-Bis(4-hydroxy-3-methoxyphenyl)-2,3-dimethyl-1-butanone
24	27.60	[M-H]^-^	355.1177	355.1187	−2.8	C_20_H_20_O_6_	356.13	6-(3,4-dihydroxy-5-methoxyphenyl)-2,3-dihydro-2-[2-(4-hydroxyphenyl)ethyl]-4H-Pyran-4-one
25	28.06	[M-H]^-^	385.1648	385.1657	−2.3	C_22_H_26_O_6_	386.17	—
26	29.62	[M-H]^-^	323.1287	323.1289	−0.6	C_20_H_20_O_4_	324.14	(4E,6E)-7-(4-Hydroxy-3-methoxyphenyl)-1-(4-hydroxyphenyl)-4,6-heptadien-3-one
27	29.81	[M-H]^-^	551.2645	551.2650	−0.9	C_32_H_40_O_8_	552.27	—
28	30.17	[M + H]^+^	445.2202	445.2221	−4.3	C_25_H_32_O_7_	444.21	(2R,3S,8R,10S)-2-(3,4-Dihydroxyphenyl)-10-heptyl-3,4,9,10-tetrahydro-2H,8H-benzo[1,2-b:3,4-b′]dipyran-3,5,8-triol
29	30.32	[M + H]^+^	318.2994	318.3003	−2.8	C_18_H_39_NO_3_	317.29	Phytosphingosine
30	34.47	[M + H]^+^	520.3394	520.3398	−0.8	C_26_H_50_NO_7_P	519.33	1-Linoleoyl-sn-glycero-3-phosphocholine
31	35.01	[M-H2O + H]^+^	299.2008	299.2006	0.7	C_20_H_28_O_3_	316.20	4-Hydroxyretinoic acid
32	35.30	[M + H]^+^	496.3393	496.3398	−1.0	C_24_H_50_NO_7_P	495.33	1-Palmitoyl-sn-glycero-3-phosphocholine
33	35.50	[M + H]^+^	273.2582	273.2577	1.8	C_20_H_32_	272.25	Gamma-camphorene
34	35.77	[M-H]^-^	295.2272	295.2279	−2.4	C_18_H_32_O_3_	296.24	13-Hydroxy-9,11-octadecadienoic acid
35	35.89	[M + H]^+^	522.3568	522.3554	2.7	C_26_H_52_NO_7_P	521.35	1-Vaccenoyl-glycero-3-phosphocholine
36	37.11	[M-H2O + H]^+^	409.3830	409.3829	0.2	C_30_H_50_O	426.39	Hop-17(21)-en-3β-ol
37	39.02	[M-H]^-^	595.2873	595.2889	−2.7	C_27_H_49_O_12_P	596.30	1-(9Z,12Z-octadecadienoyl)-glycero-3-phospho-(1′-myo-inositol)
38	39.89	[M-H]-	277.2174	277.2173	0.4	C18H30O2	278.22	Linolenic acid
39	40.35	[M + H]+	305.2468	305.2475	−2.3	C20H32O2	304.24	Arachidonic acid
40	40.51	[M-H]-	571.2885	571.2889	−0.7	C25H49O12P	572.30	1-Palmitoyl-3-glycerylphosphorylinositol
41	41.36	[M-H]-	279.2320	279.2330	−3.6	C18H32O2	280.24	Linoleic acid
42	42.03	[M + H]+	593.2752	593.2745	1.2	C35H36N4O5	592.27	Pheophorbide A

### 3.3 CG is associated with necroptosis in the treatment of UC

**FIGURE 3:**
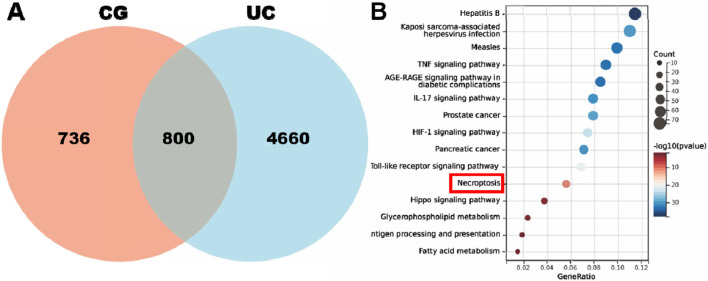
CG is associated with necroptosis in the treatment of UC. **(A)** Venn diagrams of the targets of CG and UC. **(B)** KEGG analysis.

We conducted an analysis to identify the targets of the active components of CG that were detected. Following the elimination of duplicate entries, we identified a total of 1,536 unique targets. We conducted an analysis of the “components and targets” network of CG (Supplementary Figure S1). Subsequently, we investigated the potential association between CG and the necroptosis pathway. By determining the intersection of the targets associated with CG and UC, we identified 800 common targets (Figure 3A). A KEGG pathway analysis of these 800 targets indicated that the therapeutic mechanism of CG in the treatment of UC is associated with the necroptosis pathway (Figure 3B). By intersecting these targets with necroptosis, we hypothesized that components such as catechin, phytosphingosine, and procyanidin in CG might possess potential anti-necroptotic activity. Consequently, we conducted a preliminary screening of these components to assess their anti-necroptotic activity. However, our results did not identify any active ingredients that exhibited anti-necroptotic activity (Supplementary Figure S2). Therefore, it is speculated that the anti-necroptosis activity may be attributable to the synergistic action of multiple interaction components.

### 3.4 STAT3 is a potential target for treating UC through necroptosis

**FIGURE 4:**
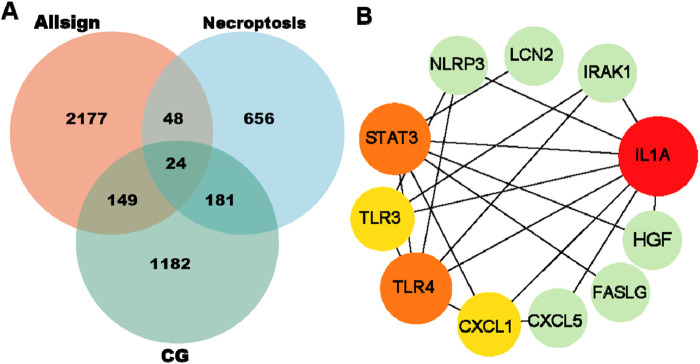
Potential target of CG for treating UC through necroptosis. **(A)** A Venn diagram depicting the intersection of differentially expressed genes from the GEO database with CG targets and necroptosis-related genes. **(B)** An analytical diagram of the PPI network for the intersecting genes.

The intersection of drug targets associated with CG, differentially expressed genes from the GEO database, and necroptosis-related genes were identified, resulting in a set of 24 genes. These genes may serve as potential drug action targets for CG in inhibiting necroptosis during the treatment of UC (Figure 4A). The PPI analysis was performed on 24 targets, prioritized according to their degree of connectivity. The findings suggested that IL1A, STAT3, TLR4, TLR3, and CXCL1 are the most probable targets for the inhibitory action of CG on necroptosis. Notably, within the network analysis, STAT3 is identified as a key hub node with direct or indirect interactions with the core effector molecules of necroptosis (RIPK1, RIPK3, MLKL), whereas IL1A, TLR4, and other molecules are predominantly enriched in upstream inflammatory signaling pathways (Shi et al., 2024). Furthermore, there is no research available on CG’s regulation of STAT3. Therefore, STAT3 may exert its therapeutic effects by inhibiting necroptosis via the modulation of STAT3 (Figure 4B).

### 3.5 CG is identified as a potential necroptosis inhibitor

**FIGURE 5:**
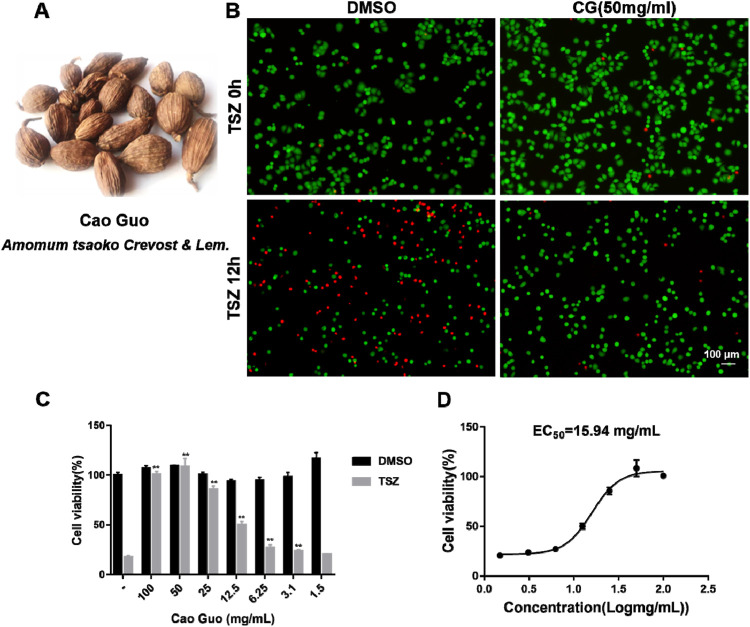
CG as a potential necroptosis inhibitor. **(A)** Image of herb CG; **(B)** Representative images of calcein-AM/PI staining of CG subjected to various treatments over a 12-h period; Green represents living cells, while red represents dead cells. Scale bar = 100 μm. **(C)** HT-29 cells were treated with a specified concentration of CG, followed by stimulation with TSZ for 12 h, after which cell viability was assessed using the CellTiter-Glo chemiluminescence assay; **(D)** The half-maximal effective concentration (EC_50_) of CG for the inhibition of necroptosis was determined. **P < 0.01 compared to TSZ group.

We performed a study to examine the anti-necroptotic effects of CG *in vitro* utilizing a TNF-α, SMAC, and z-VAD-FMK (TSZ, necroptosis inducer) induced necroptosis model in HT-29 cells. Figure 5A is the image of herb CG. Post-treatment with CG, there was a significant decrease in cell death, accompanied by a higher number of viable cells, as demonstrated by the Calcein/PI Cell Viability/Cytotoxicity Assay Kit (Figure 5B). In this assay, Calcein AM stained live cells green, whereas Propidium Iodide marked dead cells red. Furthermore, the cells were incubated with varying concentrations of CG for a duration of 12 h. CG markedly enhanced cell viability, exhibiting an EC_50_ value of 15.94 mg/mL (Figures 5C,D).

It is important to acknowledge that the concentrations utilized in this study are derived from the dry weight concentration of the crude extract, which comprises numerous potentially inactive components. As a result, the observed range of active concentrations (1.5–100 mg/mL) is significantly higher than those typically employed in conventional small molecule drug experiments. At such elevated concentrations, factors such as osmotic pressure may exert non-specific effects on cells. The interpretation of the results should be approached with caution within this context.

### 3.6 CG inhibits HT-29 cell necroptosis signaling

**FIGURE 6:**
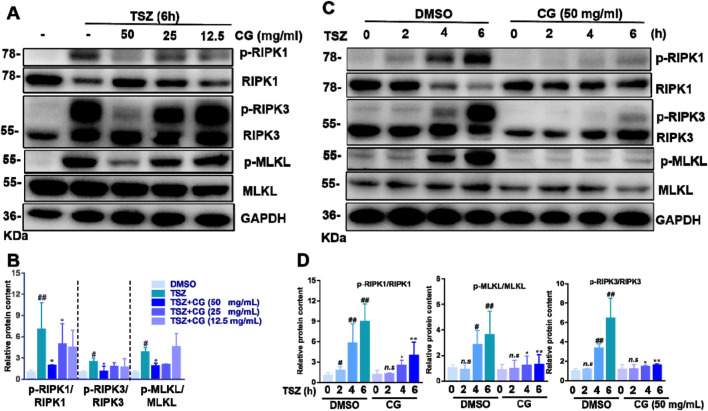
CG suppresses the activation of the necroptosis signaling pathway. **(A)** HT-29 cells were treated with varying concentrations of CG for a duration of 6 h. **(B)** A quantitative analysis was conducted on the grayscale intensities of protein bands subjected to different concentrations of CG over a 6-h period. ^##^ *P* < 0.01 and ^#^ *P* < 0.05 versus DMSO, **P < 0.01 and *P < 0.05 versus TSZ simulation. **(C)** HT-29 cells were pretreated with CG at a concentration of 50 mg/mL for 30 min, followed by exposure to TSZ for specified time intervals. **(D)** A quantitative analysis was performed on the grayscale intensities of protein bands across different time points. ^##^ *P* < 0.01 and ^#^ *P* < 0.05 versus TSZ simulation (0h), **P < 0.01 and *P < 0.05 versus TSZ simulation with the corresponding time, *n.s*, significant. Data are presented as the mean ± standard deviation. Scale bar as indicated.

RIPK1, RIPK3, and MLKL are pivotal proteins within the necroptosis signaling pathway. The phosphorylation of these proteins induces and amplifies the extent of necroptosis. Therefore, we evaluated the phosphorylation status of RIPK1, RIPK3, and MLKL in the presence of CG. Our experimental findings revealed that CG inhibited the phosphorylation of RIPK1, RIPK3, and MLKL in a dose-dependent manner (Figures 6A,B). Furthermore, CG was able to suppress the phosphorylation status of RIPK1, RIPK3, and MLKL even under continuous stimulation with TSZ, which typically enhances the activation of the necroptosis pathway (Figures 6C,D).

### 3.7 CG alleviates DSS-induced UC injury in mice

**FIGURE 7:**
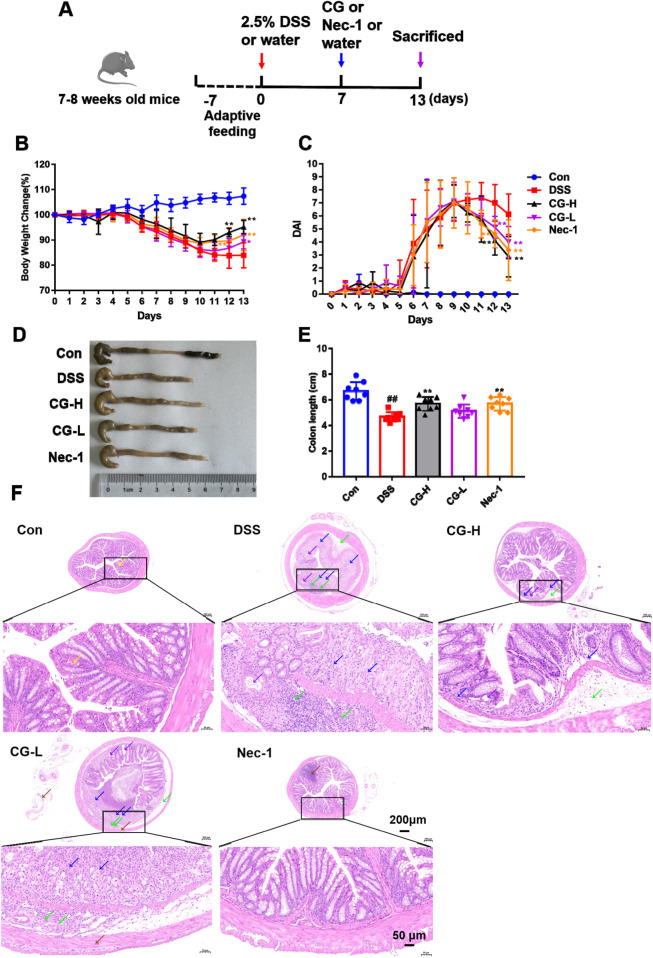
The protective effects of CG on DSS-induced UC in mice. **(A)** A schematic representation of the DSS-induced mouse model of UC and the corresponding administration protocol. **(B)** Graphical representation of the temporal variations in body weight across different groups of mice. **(C)** Graphical representation of the temporal variations in DAI across different groups of mice. Analysis using repeated measures ANOVA revealed a significant interaction effect between treatment and time (*p* < 0.01). Statistical significance is denoted as follows: *p < 0.05, **p < 0.01, when compared to the model group at the corresponding time point (simple effect analysis with Bonferroni correction); ^#^ *p* < 0.05, ^##^ *p* < 0.01, when compared to the control group at the same time point. **(D)** Representative images of mouse colon; **(E)** The colon length measured on day 13. **(F)** HE staining of representative colon tissue sections. ^##^ *P* < 0.01 vs. Con group. *P < 0.05 and **P < 0.01 vs. DSS group. Scale bar as indicated.

Building upon the gene expression characteristics associated with necrotic apoptosis and barrier function identified in the blood of patients with UC, we will subsequently investigate at the target tissue level, specifically the colon, to determine whether these pathways exhibit alterations in the DSS-induced mouse model of colitis. To investigate the protective effects of CG against UC, a dextran sulfate sodium (DSS)-induced mouse model was utilized. Figure 7A illustrates the schematic diagram of the experimental procedure. Necrostatin-1 (Nec-1), a well-established necroptosis inhibitor, served as a positive control at a dosage of 5 mg/kg. CG was administered at dosages of 0.91 g/kg (low dose) and 1.82 g/kg (high dose). The data indicated that CG significantly alleviated the symptoms of UC. Compared to the DSS group, the CG-treated groups showed notable weight recovery (Figure 7B), a decreased DAI score (Figure 7C), improved stool quality and reduced blood presence, as well as increased colon length (Figures 7D,E). The repeated measures ANOVA demonstrated a significant interaction effect between treatment and time on weight changes (F(52, 364) = 20.97, *p* < 0.001), as well as a significant main effect of time (*p* < 0.0001). These findings suggest that the trajectories of weight reduction varied among the different treatment groups over time. Subsequent simple effects analysis, adjusted using the Bonferroni correction, revealed that from the sixth day following DSS administration, the weight of the model group was significantly lower than that of the normal control group (*p* < 0.01). Moreover, high-dose CG pre-treatment significantly mitigated the DSS-induced weight loss (compared to the model group, *p* < 0.05), with this effect persisting until the conclusion of the experiment. A repeated measures analysis of variance identified a significant ‘treatment × time’ interaction effect on DAI changes (F(52, 364) = 9.021, *p* < 0.001), as well as a significant main effect of time (*p* < 0.0001). These findings suggest that the trajectory of DAI reduction over time varied across the different treatment groups of mice. Subsequent simple effects analysis, adjusted using the Bonferroni correction, revealed that from the fifth day following DSS administration, the DAI in the model group was significantly lower than that in the normal control group (*p* < 0.01). Moreover, high-dose CG pre-treatment markedly mitigated the DSS-induced decline in DAI (compared to the model group, *p* < 0.05), with this effect persisting until the conclusion of the experiment.The therapeutic effects observed in the high-dose CG group were comparable to those in the Nec-1 group. To assess the extent of intestinal injury, histopathological analysis was conducted using hematoxylin and eosin (HE) staining. As illustrated in Figure 7F, the intestinal tissues of mice in the DSS group exhibited extensive necrosis, detachment, and absence of mucosal epithelial cells. Numerous intestinal gland structures within the lamina propria were absent, having been supplanted by proliferating connective tissue, which extended into the submucosa and was associated with significant infiltration of lymphocytes and granulocytes (denoted by blue arrows). The adjacent intestinal glands displayed irregular arrangement, with occasional expansions of the intestinal glands observed (indicated by purple arrows). Infrequently, intestinal gland structures were noted within the submucosa, although dilation of these glands was rare (also indicated by purple arrows). Moderate edema was present in the submucosa, where the connective tissue appeared loosely organized, accompanied by substantial infiltration of lymphocytes and granulocytes (highlighted by green arrows). The smooth muscle cells within the muscular layer were observed to be regularly arranged. In the high-dose CG group, the intestinal tissues occasionally exhibited an absence of mucosal epithelial cells. A reduction in the number of intestinal gland structures within the lamina propria was noted, with these structures being supplanted by proliferating connective tissue, accompanied by minimal lymphocyte infiltration, as indicated by blue arrows. The surrounding intestinal glands demonstrated irregular arrangement, and occasional expansions of the intestinal glands were observed, as indicated by purple arrows. Mild edema was present in the submucosa, characterized by loosely arranged connective tissue and a small degree of lymphocyte infiltration, denoted by green arrows. The smooth muscle cells within the muscular layer maintained regular arrangement. The results revealed that CG treatment ameliorated the loss of intestinal epithelial cells, the extent of submucosal edema, and lymphocyte infiltration (Figure 7F).

### 3.8 CG alleviates intestinal inflammation and barrier function in UC mice

**FIGURE 8:**
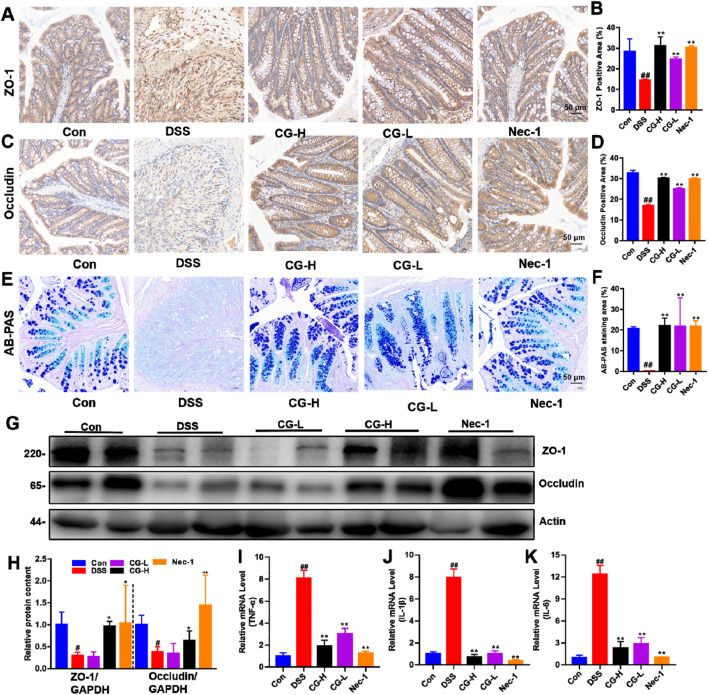
The effects of CG on intestinal inflammation and barrier function in a murine model of UC. Immunofluorescence techniques were employed to evaluate the influence of CG on the distribution and expression of ZO-1 **(A)** and Occludin **(C)** in mice with DSS-induced UC. The quantitative analyses of the fluorescence intensities of ZO-1 **(B)** and Occludin **(D)**. **(E)** The representative images of colon tissue stained with AB-PAS. **(F)** The quantitative assessment of the AB-PAS staining area. **(G)** Western blotting showing the relative protein expression of ZO-1 and Occludin. **(H)** The quantitative analysis of the grayscale intensities of protein bands in colon tissues. The impact of CG on the mRNA levels of pro-inflammatory cytokines TNF-α **(I)**, IL-1β **(J)**, and IL-6 **(K)** in colon tissue, as measured by quantitative reverse transcription PCR (qRT-PCR). ^##^ *P* < 0.01 and *^#^P* < 0.05 vs. Con group. *P < 0.05 and **P < 0.01 vs. DSS group. Scale bar as indicated.

To evaluate the effects of CG on intestinal barrier function and inflammation, we analyzed the distribution and expression of ZO-1 and Occludin and quantify intestinal goblet cells, and measured the mRNA expression levels of *TNF-α*, *IL-1β*, and *IL-6* in colonic tissues. The experimental results reveal that CG significantly enhances the expression of ZO-1 (Figures 8A,B) and Occludin-1 (Figures 8C,D). The goblet cell count, as evaluated through AB-PAS staining, indicated that CG treatment led to an increased number of goblet cells compared to the DSS model group (Figures 8E,F). Besides, the Western blot analysis indicated a significant increase in ZO-1 and Occludin-1 expression in the CG groups (Figures 8G,H). RT-PCR analysis demonstrated that CG reduced the mRNA expression levels of *TNF-α*, *IL-1β*, and *IL-6* in colonic tissues, suggesting that CG alleviates DSS-induced intestinal inflammation, as illustrated in Figures 8I–K.

### 3.9 CG alleviates the necroptosis of IECs in UC mice

**FIGURE 9:**
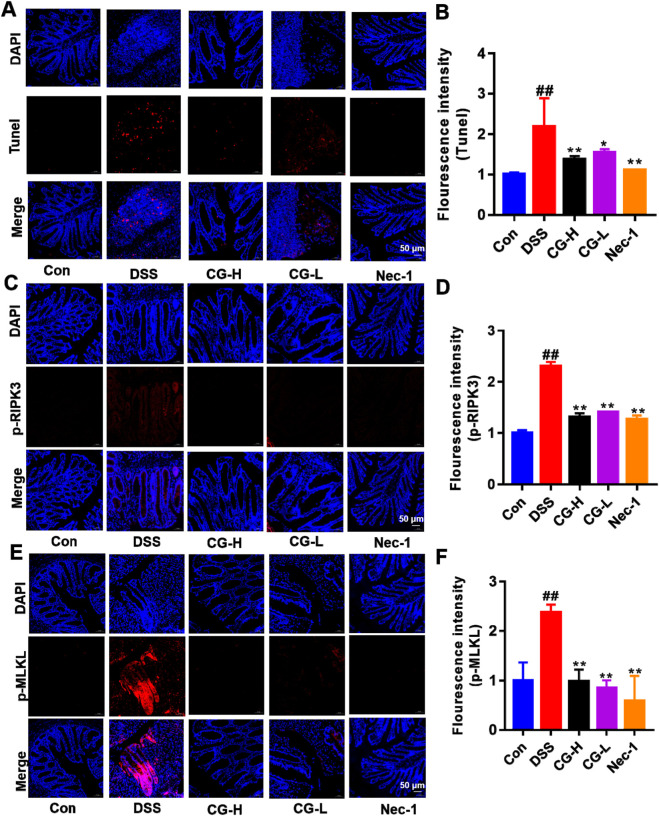
Effect of CG on necroptosis of IECs in UC mice. **(A)** Analysis of IECs death by TUNEL. **(B)** Quantitative analysis of fluorescence intensity of Tunel. **(C)** The distribution and expression levels of p-RIPK3 in colon tissues. **(D)** Quantitative analysis of fluorescence intensity of p-RIPK3. **(E)** The distribution and expression levels of p-MLKL in colon tissues. **(F)** Quantitative analysis of fluorescence intensity of p-MLKL. ^##^ *P* < 0.01 and ^#^ *P* < 0.05 vs. Con group. *P < 0.05 and **P < 0.01 vs. DSS group. Scale bar as indicated.

The TUNEL staining results demonstrated that CG mitigated cell death of intestinal epithelial cells (Figures 9A,B). Additionally, immunofluorescence analysis revealed that CG inhibited the phosphorylation levels and altered the distribution of phospho-RIPK3 (Figures 9C,D) and phospho-MLKL (Figures 9E,F) in intestinal epithelium.

### 3.10 CG inhibits phosphorylation activation of STAT3 to alleviate necroptosis of IECs

**FIGURE 10:**
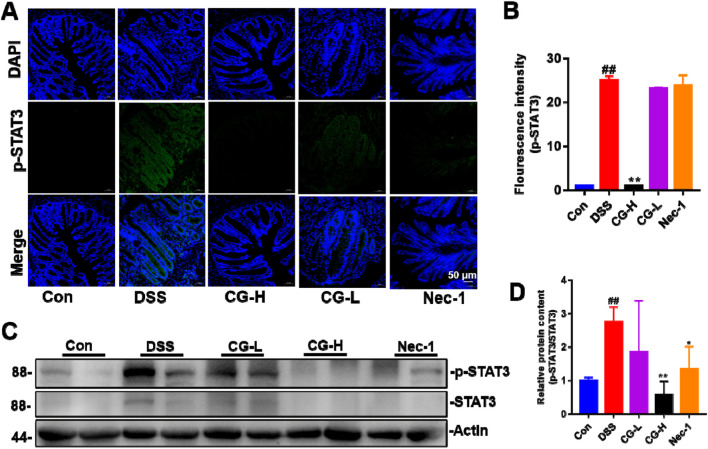
Effect of CG on phosphorylation levels and distributions of STAT3. **(A)** Effect of CG on phosphorylation levels and distributions of STAT3 in colon tissues. **(B)** Quantitative analysis of fluorescence intensity of p-STAT3. **(C)** Western blotting showing the relative protein expression of STAT3 and p-STAT3. **(D)** The quantitative analysis of the grayscale intensities of protein bands in colon tissues. ^##^ *P* < 0.01 and ^#^ *P* < 0.05 vs. Con group. *P < 0.05 and **P < 0.01 vs. DSS group. Scale bar as indicated.

To investigate the impact of CG on the phosphorylation status of STAT3, immunofluorescence techniques were employed to assess the distribution and expression levels of phosphorylated STAT3 (p-STAT3). Immunofluorescence indicates that a high dose of CG significantly suppresses the phosphorylation level and distribution of STAT3 (Figures 10A,B). Western blot analysis indicated a significant decrease in p-STAT3 expression in the high dose of CG group (Figures 10C,D). These observations suggest that CG may mitigate necroptosis in intestinal epithelial cells by inhibiting STAT3 phosphorylation.

### 3.11 CG improves necroptosis via STAT3 in HT-29 cells

**FIGURE 11:**
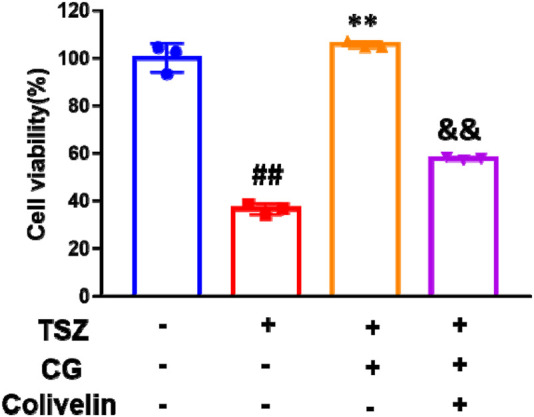
UA improves necroptosis via STAT3 *in vitro*. Cell activity of CG in the presence and absence of colivelin in TSZ induced HT-29 cell. ^##^ *P* < 0.01 compared to DMSO group. **P < 0.01 compared to TSZ group. ^&&^ *P* < 0.01 compared to CG group.

To determine if STAT3 is a target for modulating CG’s inhibition of necroptosis, we assessed HT-29 cell viability with TSZ, with and without the STAT3 agonist colivelin. The result indicated that colivelin diminished the protective effect of CG against TSZ-induced necroptosis (Figure 11).

## 4 Discussion

UC is a chronic inflammatory disorder with a global prevalence of approximately 5 million individuals as of 2023 (Le Berre et al., 2023; Gobert et al., 2022). The incidence of UC continues to rise worldwide, underscoring the urgent need for novel and effective therapeutic interventions. TCM, with its extensive historical use in China, has garnered increasing attention in various medical domains due to its low toxicity and high efficacy. It has been extensively applied in the treatment of numerous diseases (Shu et al., 2020)。In recent years, there has been significant global interest in herbs that serve dual purposes as both food and medicine, such as citrus fruits (Da Pozzo et al., 2018) and yam (Khol et al., 2024). Cao Guo, a staple in both traditional Chinese medicine and culinary practices, is recognized for its therapeutic properties in treating conditions like malaria, abdominal pain, and vomiting. Our research indicates that CG ameliorates symptoms of DSS-induced colitis in mice, including bloody stools, diarrhea, and weight loss, while also mitigating colonic pathological damage.

Inhibition of necroptosis in intestinal epithelial cells has been demonstrated to mitigate cell loss and enhance intestinal barrier function, thereby contributing to the treatment of UC (Zhang et al., 2020; Lee et al., 2020). Our study, through analysis of genomic data from the GEO database, identified a strong association between UC and the pathological mechanism of necroptosis. Necroptosis is a form of programmed cell death that occurs independently of caspases, with its key mediators being the proteins RIPK1, RIPK3, and MLKL (Song et al., 2022). Currently, small molecule inhibitors targeting RIPK1, RIPK3, and MLKL have been developed. To date, over 40 types of RIPK1 inhibitors have been synthesized. However, their clinical trials have largely not progressed beyond phase II. Similarly, although RIPK3 inhibitors have been investigated, their clinical application is constrained by significant cytotoxicity. Furthermore, the development of MLKL inhibitors is limited due to challenges such as poor specificity and high cytotoxicity, resulting in a relatively small number of available MLKL inhibitors. To date, no compounds targeting the three primary targets of necroptosis have been approved for clinical use (Gardner et al., 2023; Xu and Zhuang, 2023; Zhuang and Chen, 2020; Zou et al., 2025). Our research demonstrated that in the TSZ-induced necroptosis model of HT-29 cells, CG enhanced cellular morphology and improved cell viability in a dose-dependent manner, with an EC_50_ value of 15.94 mg/mL. In the DSS-induced mouse model of ulcerative colitis, CG was found to enhance the expression of mechanical barrier proteins ZO-1 and Occludin. In the TUNEL assay, CG reduces the loss of intestinal epithelial cells. Furthermore, CG inhibited the expression and distribution of phosphorylated RIPK3 and MLKL in colon tissues.

We analyzed the chemical composition of CG and conducted intersections with differentially expressed genes from the GEO database, as well as with the targets associated with both the chemical composition of CG and necroptosis. Among these targets, STAT3 emerged as a potential target through which CG may inhibit necroptosis. The role of STAT family proteins in the pathogenesis of inflammatory bowel disease (IBD) is a subject of ongoing investigation. STAT3, a latent cytoplasmic protein expressed across various metabolic tissues, is regulated through multiple post-translational modifications. Phosphorylation at tyrosine-705 is a critical event that induces STAT3 dimerization and facilitates its translocation to the nucleus (Soutto et al., 2019). STAT3 is implicated in the regulation of numerous cellular processes, including cell growth, proliferation, differentiation, survival, apoptosis, and angiogenesis (Kleinbongard, 2023). Several studies have documented elevated levels of STAT3 or its phosphorylated form in human IBD cases (Mudter et al., 2005; Musso et al., 2005). Evidence from IBD patients underscores the role of STAT3 activation, primarily through IL-6 signaling, in conferring resistance to apoptosis in T cells (Atreya et al., 2000). Moreover, findings from murine models corroborate the pathogenic role of STAT3. Specifically, mice possessing with a conditional knockout that circumvents the inhibitory effects of cytokine signal transduction inhibitor 3 (SOCS3) exhibit excessive STAT3 activation, leading to severe colitis (Suzuki et al., 2001). Conversely, inhibition of STAT3-induced apoptosis in the lamina propria ameliorates colitis (Bai et al., 2007). Moreover, a study has demonstrated that ursolic acid may suppressed necroptosis by inhibiting the phosphorylation of STAT3, thereby offering a therapeutic approach for intestinal ischemia-reperfusion injury (Shi et al., 2024). Consequently, STAT3 emerges as a potential target for the inhibition of necroptosis. Our research suggests that CG can suppress the expression and distribution of phosphorylated STAT3 levels, indicating that it may serve as a potential target for CG in inhibiting necroptosis in the treatment of UC.

A limitation of this study is that the GEO data utilized for bioinformatics analysis were derived from blood samples. While the blood transcriptome can effectively reflect the systemic immune-inflammatory state of the disease and provides significant candidate pathways, its correlation with the local mucosa of the colon, particularly the specific mechanisms of intestinal epithelial cells, is indirect. Consequently, the immunofluorescence (Figure 9) and barrier marker detection (Figure 8) performed on colon tissue in the animal model serve as functional validations and extended explorations of these systemic clues in the target tissue, rather than direct evidence. Future research should aim to directly verify these findings in colon biopsy tissue samples from UC patients.

However, while our network pharmacology analysis offers a systematic prediction of the mechanism underlying CG’s action, it fundamentally relies on computational inference derived from correlations within public databases. The hub genes identified, such as STAT3, and the associated pathways require functional gain/loss experiments *in vivo* to validate their specific roles and regulatory directions in the alleviation of UC by CG.

**FIGURE 12:**
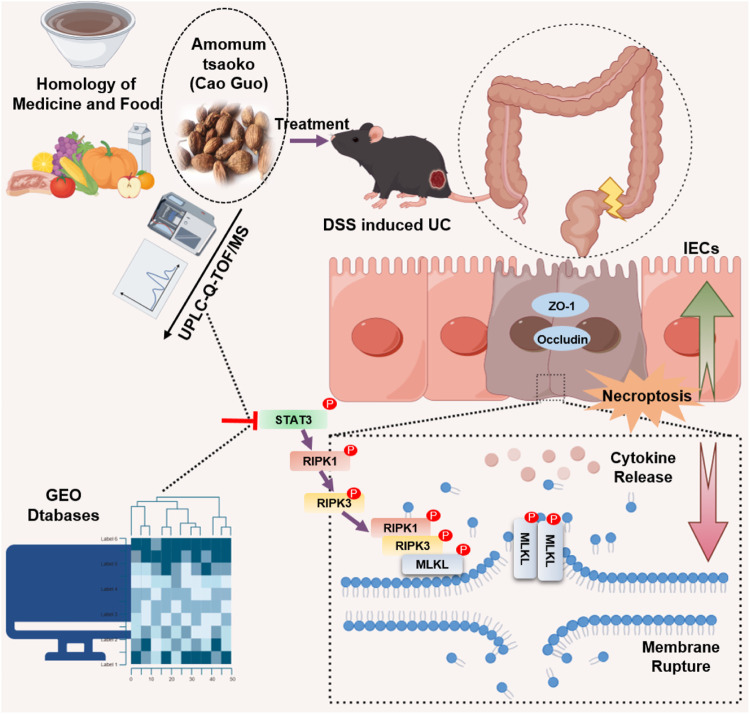
Diagrammatic depiction of proposed mechanism of CG on the protective effect of UC.

In conclusion, our analysis of the GEO database indicates a significant association between UC and necroptosis. Both *in vivo* and *in vitro* experiments confirmed that CG exhibits potential in inhibiting necroptosis. Additionally, through an examination of the potential targets of CG’s chemical constituents and bioinformatics analysis, we determined that CG may inhibit necroptosis by reducing the phosphorylation level of STAT3. These results elucidate a novel mechanism of action for CG in the treatment of UC and provide a scientific foundation for the use of Chinese medicine as an inhibitor of necroptosis. By focusing on the modulation of STAT3 to regulate necroptosis in intestinal epithelial cells, this method presents a promising therapeutic strategy warranting further investigation for UC and other diseases associated with necroptosis. The molecular mechanism by which CG exerts its therapeutic effects on UC is depicted in Figure 12.

## Data Availability

The original contributions presented in the study are included in the article/Supplementary Material, further inquiries can be directed to the corresponding authors.
