# Supplementary material for: Amomum tsaoko extract from Nujiang alleviates DSS-induced colitis through inhibiting necroptosis
Source: Front Cell Dev Biol. 2026 Feb 26;14:1768630. doi: 10.3389/fcell.2026.1768630 (PMC12979518; doi:10.3389/fcell.2026.1768630)
Supplement: Supplementary file 2 [file DataSheet1.docx]

*Amomum tsaoko* Extract from Nujiang Alleviates DSS-Induced Colitis through Inhibiting Necroptosis

Yuanyuan Wang^ab^, Keyi Lu^ab^, Yuhang Gong^ab^, Yanna Shao^ab^, Siqi Liu^ab^, Yuan Fang^e^, Yifan Shi^ab^,Erping Xu^ab^, Yanqiong Yang^c^, Si Yuan ^c^, Ming Bai^ab^*, Zhibin Wang^d^*, Bo Zhang^e^*

^a^ Collaborative Innovation Center of Research and Development on The Whole Industry Chain of Yu-Yao, Henan Province, Henan University of Chinese Medicine, Zhengzhou, Henan 450046, P.R. China;

^b^ Academy of Chinese Medical Sciences, Henan University of Chinese Medicine, Zhengzhou, Henan 450046, P.R. China;

^c^ Ningjiang Lisui Autonomous Prefecture Traditional Chinese Medicine Hospital

^d^ Department of Critical Care Medicine, School of Anesthesiology, Naval Medical University, Shanghai, China.

^e^ Shanghai Municipal Hospital of Traditional Chinese Medicine, Shanghai University of Traditional Chinese Medicine, Shanghai 200071, P.R. China.

**AUTHOR INFORMATION**

**Corresponding Author**

*E-mail: [bob229@163.com](mailto:bob229@163.com) (Bo Zhang);

methyl@smmu.edu.cn (Zhibin Wang);

[baiming666@163.com](mailto:baiming666@163.com) (Ming Bai)；

**Notes**

The authors declare no competing financial interests.

**<Supporting Information>**

1. **Supplement Figure 1**


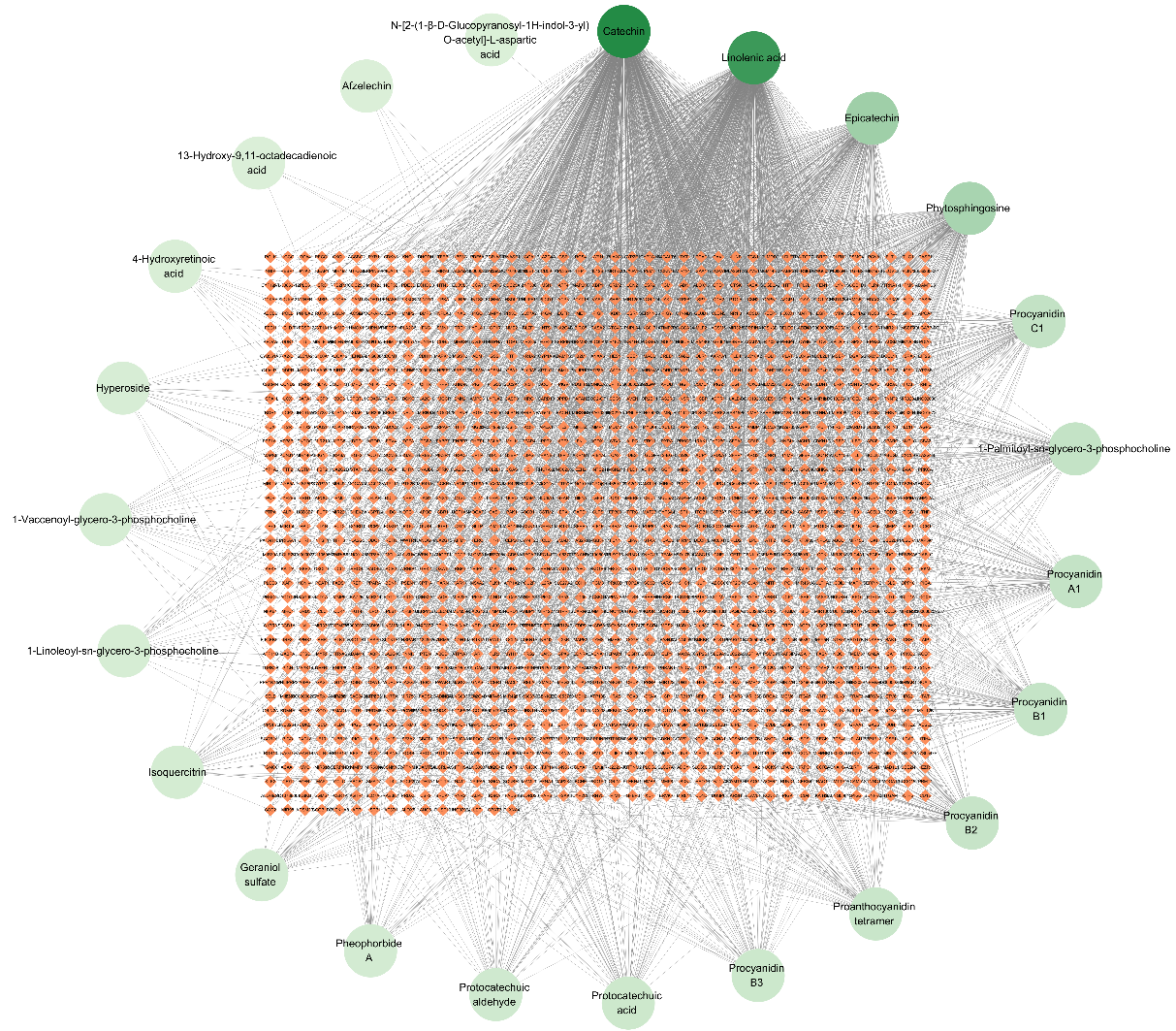


**Supplement Figure 1**

The analysis of the ‘components and targets’ network of CG.

1. **Supplement Figure 2**

**
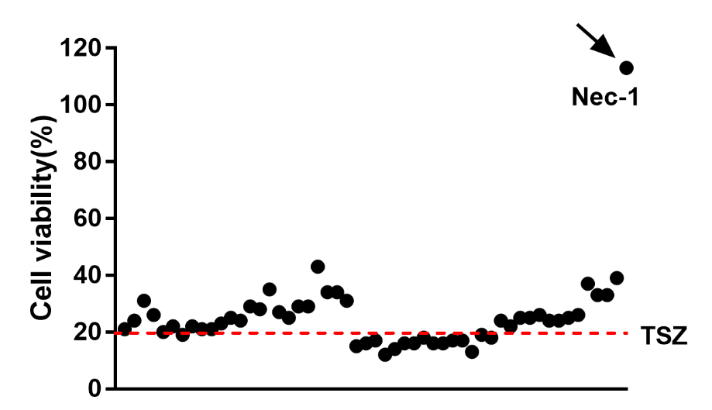
**

**Supplement Figure 2**

Screening compounds of CG to evaluate their activity against necroptosis. The primary screening concentration of all compounds was 10 μM.

**3. The Full Bolts of Western Blots**


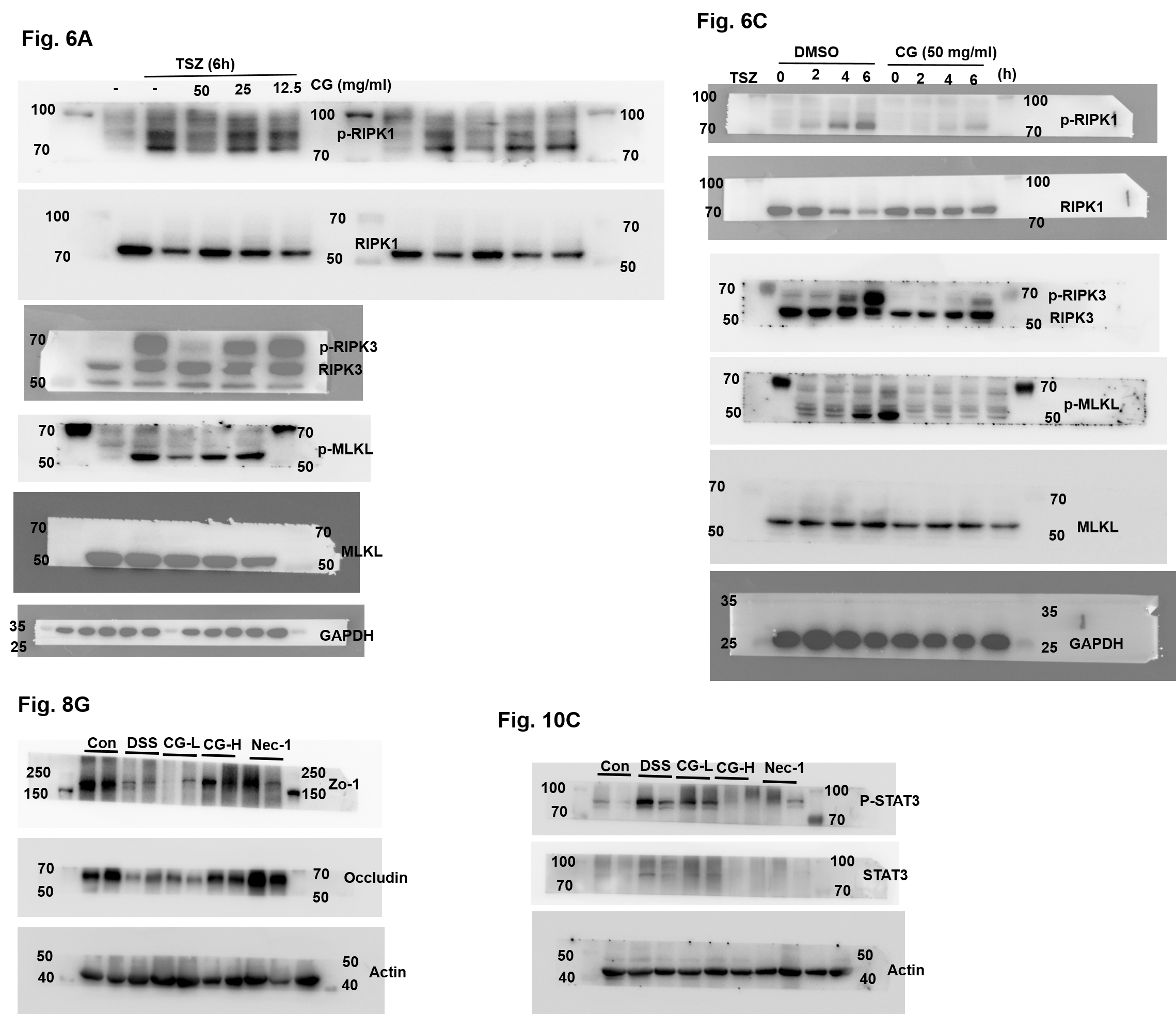


**4. Calcein-AM/PI staining**


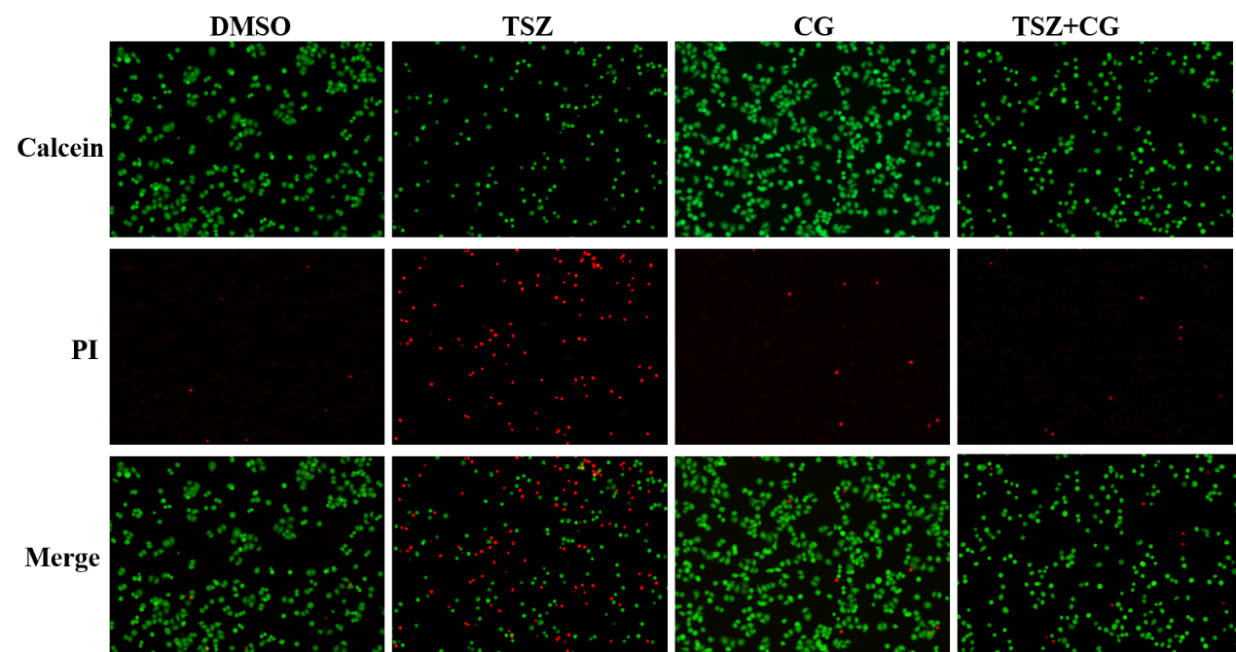


5. The original version of the relevant images.


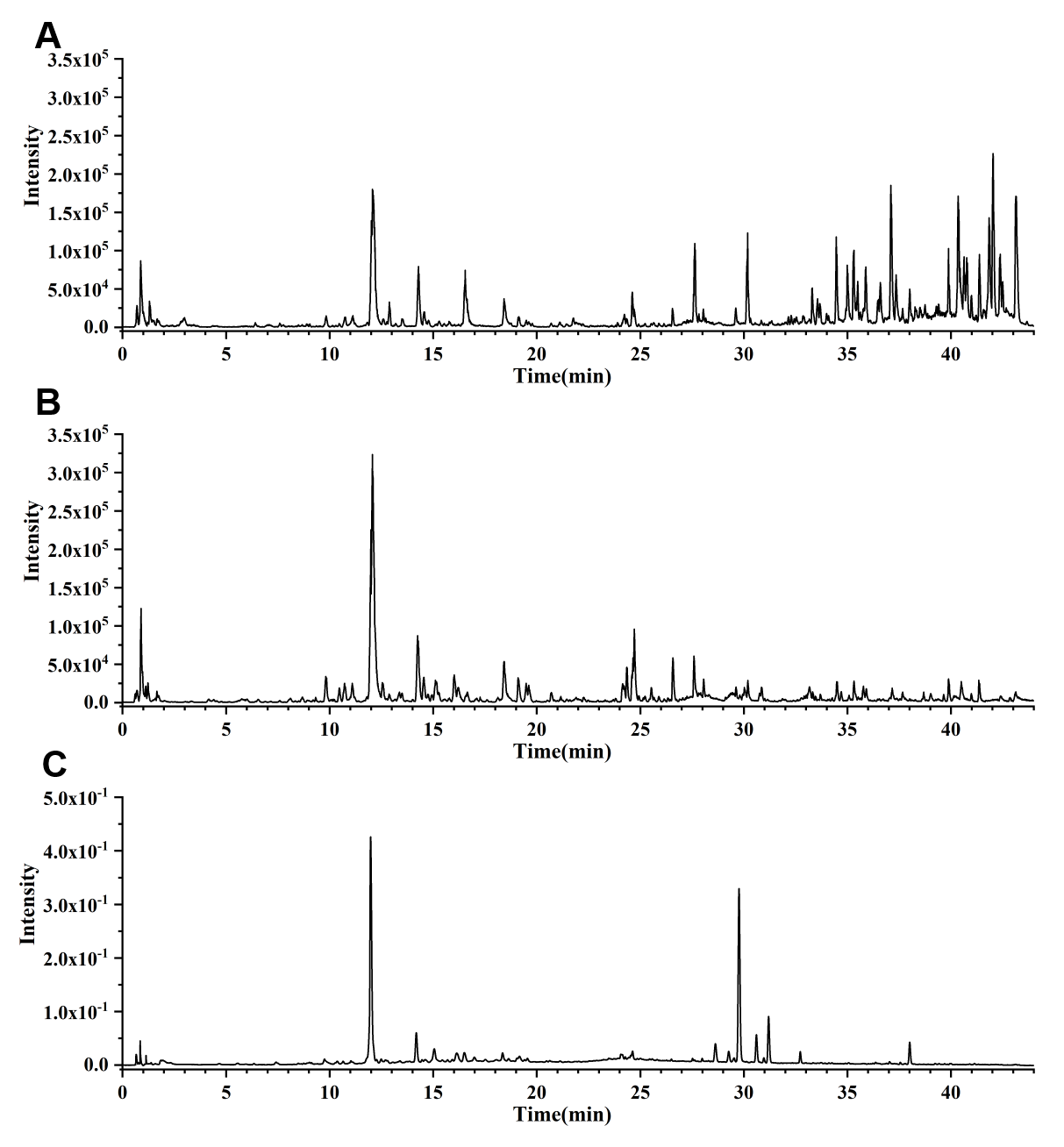


(A) Positive ion mode UPLC-HRMS base peak ion flow graph (BPC) for CG; (B) Negative ion mode UPLC-HRMS BPC for CG; (C) UPLC UV chromatogram of CG at 280 nm.

**Supplement Table S1** Identification results of components of CG.

| NO. | Time（min） | **Adducts** | **Measured** *m/z* | Excepted *m/z* | ppm | Formula | Molecular Weight | name | MS/MS | peak area |
| --- | --- | --- | --- | --- | --- | --- | --- | --- | --- | --- |
| 1 | 5.56 | [M-H]^-^ | 153.0188 | 153.0188 | 0.0 | C_7_H_6_O_4_ | 154.03 | Protocatechuic acid | 153.0151;109.0283;108.0219;91.0180 | 68046 |
| 2 | 7.49 | [M-H]^-^ | 137.0247 | 137.0244 | 2.2 | C_7_H_6_O_3_ | 138.03 | Protocatechuic aldehyde | 137.0228;108.0242;91.0203;81.0357;62.0230 | 109926 |
| 3 | 9.82 | [M-H]^-^ | 289.0704 | 289.0712 | -2.8 | C_15_H_14_O_6_ | 290.08 | Catechin | 289.0714;245.0799;203.0701;123.0442;109.0291 | 352997 |
| 4 | 10.47 | [M-H]^-^ | 451.1340 | 451.1340 | 0.0 | C_20_H_24_N_2_O_10_ | 452.14 | N-[2-(1-β-D-Glucopyranosyl-1H-indol-3-yl)acetyl]-L-aspartic acid | 451.1359;433.1229;407.1465;335.1234;292.1187;173.0722;132.0303;88.0402 | 163690 |
| 5 | 11.99 | [M-H]^-^ | 577.1328 | 577.1352 | -4.2 | C_30_H_26_O_12_ | 578.14 | Procyanidin B1 | 577.1349;425.0876;407.0776;289.0711;245.0818;125.0244 | 1985464 |
| 6 | 12.07 | [M-H]^-^ | 289.0716 | 289.0712 | 1.4 | C_15_H_14_O_6_ | 290.08 | (-)-Epicatechin | 289.0741;245.0797;203.0714;125.0259;123.0441;109.0309 | 4201659 |
| 7 | 14.26 | [M-H]^-^ | 865.1996 | 865.1985 | 1.3 | C_45_H_38_O_18_ | 866.21 | Procyanidin C1 | 865.1987;739.1670;695.1410;577.1357;425.0870;407.0769;289.0710;287.0555;125.0244 | 862036 |
| 8 | 14.55 | [M-H]^-^ | 273.0764 | 273.0768 | -1.5 | C_15_H_14_O_5_ | 274.08 | Afzelechin | 273.0767;255.0616;205.0875;137.0236;83.0136 | 346133 |
| 9 | 15.13 | [M-2H]^2-^ | 576.1287 | 576.1273 | 2.4 | C_60_H_50_O_24_ | 1154.27 | Proanthocyanidin tetramer | 407.0737;289.0687;243.0282;161.0234;125.0234 | 379981 |
| 10 | 16.02 | [M-H]^-^ | 233.0849 | 233.0853 | -1.7 | C_10_H_18_O_4_S | 234.09 | Geraniol sulfate | 223.0843;80.9648;79.9574 | 424542 |
| 11 | 16.54 | [M+H]^+^ | 597.3274 | 597.3269 | 0.8 | C_31_H_48_O_11_ | 596.32 | / | 597.3296;421.2840;347.1976;333.1809;234.1123;177.0547 | 878831 |
| 12 | 18.43 | [M-H]^-^ | 577.1340 | 577.1352 | -2.1 | C_30_H_26_O_12_ | 578.14 | Procyanidin B2 | 577.1391;451.1049;425.0905;407.0795;289.0730;245.0815;161.0249;125.0246 | 701459 |
| 13 | 19.12 | [M-H]^-^ | 463.0873 | 463.0882 | -1.9 | C_21_H_20_O_12_ | 464.10 | Hyperoside | 463.0868;301.0336;300.0260;271.0234;255.0297 | 309780 |
| 14 | 19.49 | [M-H]^-^ | 463.0883 | 463.0882 | 0.2 | C_21_H_20_O_12_ | 464.10 | Isoquercitrin | 463.0864;300.0252;271.0233;255.0277 | 231354 |
| 15 | 19.62 | [M-H]^-^ | 605.1666 | 605.1665 | 0.2 | C_32_H_30_O_12_ | 606.17 | [2R-[2α,3β,8(2R*,3S*)]]-8,8′-ethylidenebis[2-(3,4-dihydroxyphenyl)-3,4-dihydro-2H-1-Benzopyran-3,5,7-triol | 605.1651;453.1197;315.0871;289.0707;245.0813;205.0494 | 188526 |
| 16 | 20.71 | [M-H]^-^ | 577.1347 | 577.1352 | -0.9 | C_30_H_26_O_12_ | 578.14 | Procyanidin B3 | 577.1366;425.0870;289.0723;245.0826;205.0501;161.0240;125.0242 | 118502 |
| 17 | 22.48 | [M-H]^-^ | 477.1025 | 477.1039 | -2.9 | C_22_H_22_O_12_ | 478.11 | Isorhamnetin-3-O-glucoside | 477.1032;314.0429;300.0277;285.0394;271.0243;257.0460;243.0297 | 38475 |
| 18 | 24.23 | [M-H]^-^ | 605.1664 | 605.1665 | -0.2 | C_32_H_30_O_12_ | 606.17 | (2R,2′R,3R,3′R)-8,8′-ethylidenebis[2-(3,4-dihydroxyphenyl)-3,4-dihydro-2H-1-Benzopyran-3,5,7-triol | 605.1678;453.1193;315.0867;289.0711;271.0970;245.0816 | 142183 |
| 19 | 24.35 | [M-H]^-^ | 315.1600 | 315.1602 | -0.6 | C_19_H_24_O_4_ | 316.17 | (+)-Hannokinol | 315.1597;163.0758;149.0604;121.0653;106.0429;93.0344 | 348655 |
| 20 | 24.61 | [M+H]^+^ | 577.1350 | 577.1341 | 1.6 | C_30_H_24_O_12_ | 576.13 | Procyanidin A1 | 577.1354;425.0868;245.0443;181.0490;151.0389 | 317968 |
| 21 | 24.71 | [M-H]^-^ | 315.1594 | 315.1602 | -2.5 | C_19_H_24_O_4_ | 316.17 | 3,5-Dihydroxy-1,7-bis(4-hydroxyphenyl)heptane | 315.1586;163.0759;149.0602;121.0659;106.0420;93.0341 | 745697 |
| 22 | 25.54 | [M-H]^-^ | 313.1442 | 313.1445 | -1.0 | C_19_H_22_O_4_ | 314.15 | (5S)-5-Hydroxy-1,7-bis(4-hydroxyphenyl)-3-heptanone | 313.1433;207.1028;163.0790;149.0612;119.0502 | 156336 |
| 23 | 26.58 | [M-H]^-^ | 343.1557 | 343.1551 | 1.7 | C_20_H_24_O_5_ | 344.16 | rel-(2R,3S)-1,4-Bis(4-hydroxy-3-methoxyphenyl)-2,3-dimethyl-1-butanone | 343.1532;328.1297;177.0554;161.0601;137.0240 | 443173 |
| 24 | 27.60 | [M-H]^-^ | 355.1177 | 355.1187 | -2.8 | C_20_H_20_O_6_ | 356.13 | 6-(3,4-dihydroxy-5-methoxyphenyl)-2,3-dihydro-2-[2-(4-hydroxyphenyl)ethyl]-4H-Pyran-4-one | 355.1170;340.0939;249.0756;234.0527;179.0342;164.0112;136.0161 | 456873 |
| 25 | 28.06 | [M-H]^-^ | 385.1648 | 385.1657 | -2.3 | C_22_H_26_O_6_ | 386.17 | / | 385.1671;367.1557;307.1338;217.0876;201.0926;186.0686;159.0446;59.0139 | 223290 |
| 26 | 29.62 | [M-H]^-^ | 323.1287 | 323.1289 | -0.6 | C_20_H_20_O_4_ | 324.14 | (4E,6E)-7-(4-Hydroxy-3-methoxyphenyl)-1-(4-hydroxyphenyl)-4,6-heptadien-3-one | 323.1279;308.1027;217.0866;201.0557;188.0472;159.0452;149.0605;134.0605 | 160739 |
| 27 | 29.81 | [M-H]^-^ | 551.2645 | 551.2650 | -0.9 | C_32_H_40_O_8_ | 552.27 | / | 551.2655;383.1487;323.1284;217.0862;202.0621;145.0659 | 98010 |
| 28 | 30.17 | [M+H]^+^ | 445.2202 | 445.2221 | -4.3 | C_25_H_32_O_7_ | 444.21 | (2R,3S,8R,10S)-2-(3,4-Dihydroxyphenyl)-10-heptyl-3,4,9,10-tetrahydro-2H,8H-benzo[1,2-b:3,4-b′]dipyran-3,5,8-triol | 445.2220;427.2095;401.1943;329.1005;303.0849;249.1469;177.0533;151.0380;147.0438;123.0429 | 197742 |
| 29 | 30.32 | [M+H]^+^ | 318.2994 | 318.3003 | -2.8 | C_18_H_39_NO_3_ | 317.29 | Phytosphingosine | 318.3007;300.2900;282.2900;270.2791;60.0437 | 84288 |
| 30 | 34.47 | [M+H]^+^ | 520.3394 | 520.3398 | -0.8 | C_26_H_50_NO_7_P | 519.33 | 1-Linoleoyl-sn-glycero-3-phosphocholine | 520.3396;502.3286;184.0721;124.9977;104.1054;86.0944 | 756958 |
| 31 | 35.01 | [M-H2O+H]^+^ | 299.2008 | 299.2006 | 0.7 | C_20_H_28_O_3_ | 316.20 | 4-Hydroxyretinoic acid | 299.2002;281.1904;173.1326;131.0852;97.0642;79.0536 | 650697 |
| 32 | 35.30 | [M+H]^+^ | 496.3393 | 496.3398 | -1.0 | C_24_H_50_NO_7_P | 495.33 | 1-Palmitoyl-sn-glycero-3-phosphocholine | 496.3421;478.3315;184.0735;124.9995;104.1063;86.0956 | 764233 |
| 33 | 35.50 | [M+H]^+^ | 273.2582 | 273.2577 | 1.8 | C_20_H_32_ | 272.25 | gamma-Camphorene | 273.2564;217.1948;203.1786;189.1628;161.1314;133.1001;95.0839;81.0685 | 371953 |
| 34 | 35.77 | [M-H]^-^ | 295.2272 | 295.2279 | -2.4 | C_18_H_32_O_3_ | 296.24 | 13-Hydroxy-9,11-octadecadienoic acid | 295.2267;277.2161;195.1378;171.1019 | 208528 |
| 35 | 35.89 | [M+H]^+^ | 522.3568 | 522.3554 | 2.7 | C_26_H_52_NO_7_P | 521.35 | 1-Vaccenoyl-glycero-3-phosphocholine | 522.3603;504.3495;184.0729;104.1062;86.0950 | 568612 |
| 36 | 37.11 | [M-H2O+H]^+^ | 409.3830 | 409.3829 | 0.2 | C_30_H_50_O | 426.39 | Hop-17(21)-en-3β-ol | 409.3829;327.3051;299.2765;271.2437;257.2268;231.2125;217.2125;189.1645;149.1328;121.1008;109.1007;95.0849 | 1222750 |
| 37 | 39.02 | [M-H]^-^ | 595.2873 | 595.2889 | -2.7 | C_27_H_49_O_12_P | 596.30 | 1-(9Z,12Z-octadecadienoyl)-glycero-3-phospho-(1'-myo-inositol) | 595.2884;415.2267;315.0480;279.2322;241.0108;152.9953 | 116144 |
| 38 | 39.89 | [M-H]^-^ | 277.2174 | 277.2173 | 0.4 | C_18_H_30_O_2_ | 278.22 | Linolenic acid | 277.2165;259.2043;233.2216;205.1595 | 246890 |
| 39 | 40.35 | [M+H]^+^ | 305.2468 | 305.2475 | -2.3 | C_20_H_32_O_2_ | 304.24 | Arachidonic acid | 305.2457;287.2348;203.1428;175.1473;131.0849;113.0951;105.0690;93.0689;85.1004 | 699931 |
| 40 | 40.51 | [M-H]^-^ | 571.2885 | 571.2889 | -0.7 | C_25_H_49_O_12_P | 572.30 | 1-Palmitoyl-3-glycerylphosphorylinositol | 571.2878;391.2259;315.0474;255.2326;241.0115;152.9958 | 271410 |
| 41 | 41.36 | [M-H]^-^ | 279.2320 | 279.2330 | -3.6 | C_18_H_32_O_2_ | 280.24 | Linoleic acid | 279.2310;96.9596 | 216018 |
| 42 | 42.03 | [M+H]^+^ | 593.2752 | 593.2745 | 1.2 | C_35_H_36_N_4_O_5_ | 592.27 | Pheophorbide A | 593.2783;533.2558;505.2558;460.2261 | 1787667 |
